# Di-μ-cyanido-1:2κ^2^ *C*:*N*,2:3κ^2^ *N*:*C*-hexa­cyanido-1κ^3^ *C*,3κ^3^ *C*-tetra­kis(1,10-phenanthroline)-1κ^2^ *N*,*N*′;2κ^4^ *N*,*N*′;3κ^2^ *N*,*N*′-1,3-dicobalt(III)-2-iron(II) tetra­hydrate

**DOI:** 10.1107/S1600536809030165

**Published:** 2009-08-08

**Authors:** Ying Zhang, Ai-Hua Yuan, Hu Zhou, Ji-Xi Guo, Lang Liu

**Affiliations:** a School of Material Science and Engineering, Jiangsu University of Science and Technology, Zhenjiang 212003, People’s Republic of China; b Institute of Applied Chemistry, Xinjiang University, Urumqi 830046, Xinjiang, People’s Republic of China

## Abstract

The hydro­thermal reaction of CoCl_2_·6H_2_O, 1,10-phenanthroline (phen) and K_3_[Fe(CN)_6_] in deionized water yielded the title cyanide-bridged trinuclear cluster, [Co_2_Fe(CN)_8_(C_12_H_8_N_2_)_4_]·4H_2_O or [{Co^III^(phen)(CN)_4_}_2_{Fe^II^(phen)_2_}]·4H_2_O, which contains two Co^III^ centers and one Fe^II^ center linked by cyanide bridges. The combination of coordinative bonds, O—H⋯N and O—H⋯O hydrogen bonds and π–π stacking inter­actions [centroid–centroid distance = 3.630 (2) Å] results in the stabilization of a supra­molecular structure. All uncoordinated water molecules are disordered. Thermogravimetric analysis reveals that the title complex loses the four crystal water mol­ecules at about 333 K, then the anhydrous phase loses no further mass up to about 573 K, above which decomposition occurs.

## Related literature

For background to cyanide-bridged complexes, see: Rodríguez-Diéguez *et al.* (2007 ▶); Colacio *et al.* (2003 ▶, 2005 ▶); Chen *et al.* (2006 ▶); Ferlay *et al.* (1995 ▶); Fernández-Armas *et al.* (2007 ▶); Goodwin *et al.* (2008 ▶); He *et al.* (2005 ▶); Kosaka *et al.* (2009 ▶); Mao *et al.* (2005 ▶); Overgaard *et al.* (2004 ▶); Paredes-García *et al.* (2006 ▶); Phillips *et al.* (2008 ▶); Reguera Balmaseda, del Castillo *et al.* (2008 ▶); Reguera, Balmaseda, Krap *et al.* (2008 ▶); Rodriguez *et al.* (2005 ▶); Xie *et al.* (2007 ▶); Yu *et al.* (2003 ▶). For related structures, see: Halbauer *et al.* (2008 ▶); Guo *et al.* (2007 ▶); Zhao *et al.* (2008 ▶); Brewer *et al.* (2007 ▶).
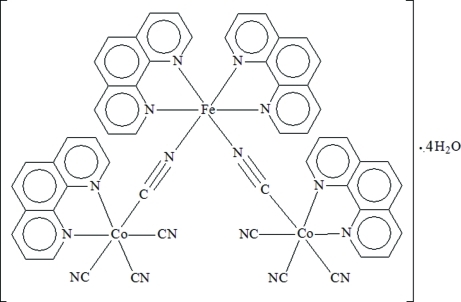


## Experimental

### 

#### Crystal data

[Co_2_Fe(CN)_8_(C_12_H_8_N_2_)_4_]·4H_2_O
*M* *_r_* = 1174.75
Triclinic, 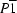

*a* = 12.855 (3) Å
*b* = 14.006 (3) Å
*c* = 16.334 (3) Å
α = 72.68 (3)°
β = 82.54 (3)°
γ = 65.99 (3)°
*V* = 2564.5 (12) Å^3^
*Z* = 2
Mo *K*α radiation
μ = 0.98 mm^−1^
*T* = 173 K
0.74 × 0.56 × 0.33 mm

#### Data collection

Rigaku R-AXIS Spider diffractometer
Absorption correction: multi-scan (*ABSCOR*; Higashi, 1995 ▶) *T*_min_ = 0.555, *T*_max_ = 0.755
41118 measured reflections
11723 independent reflections
10898 reflections with *I* > 2σ(*I*)
*R*_int_ = 0.040

#### Refinement

*R*[*F* ^2^ > 2σ(*F* ^2^)] = 0.031
*wR*(*F* ^2^) = 0.079
*S* = 1.03
11723 reflections
726 parameters
H-atom parameters constrained
Δρ_max_ = 0.44 e Å^−3^
Δρ_min_ = −0.70 e Å^−3^

### 

Data collection: *RAPID-AUTO* (Rigaku, 2004 ▶); cell refinement: *RAPID-AUTO*; data reduction: *RAPID-AUTO*; program(s) used to solve structure: *SHELXS97* (Sheldrick, 2008 ▶); program(s) used to refine structure: *SHELXL97* (Sheldrick, 2008 ▶); molecular graphics: *SHELXTL* (Sheldrick, 2008 ▶) and *DIAMOND* (Brandenburg, 2006 ▶); software used to prepare material for publication: *SHELXL97*.

## Supplementary Material

Crystal structure: contains datablocks I, global. DOI: 10.1107/S1600536809030165/at2833sup1.cif

Structure factors: contains datablocks I. DOI: 10.1107/S1600536809030165/at2833Isup2.hkl

Additional supplementary materials:  crystallographic information; 3D view; checkCIF report

## Figures and Tables

**Table 1:** Selected geometric parameters (Å, °)

Co1—C6	1.8747 (16)
Co1—C7	1.8779 (18)
Co1—C2	1.8960 (16)
Co1—C8	1.9076 (17)
Co1—N15	1.9693 (13)
Co1—N16	1.9762 (15)
Co2—C3	1.8744 (17)
Co2—C4	1.8822 (17)
Co2—C5	1.8975 (17)
Co2—C1	1.9044 (16)
Co2—N13	1.9652 (15)
Co2—N14	1.9665 (14)
Fe1—N2	2.0365 (14)
Fe1—N1	2.0464 (15)
Fe1—N12	2.0821 (15)
Fe1—N10	2.0845 (16)
Fe1—N11	2.0960 (16)
Fe1—N9	2.1067 (16)
N1—C1	1.144 (2)
N2—C2	1.144 (2)
N3—C3	1.147 (2)
N4—C4	1.147 (2)
N5—C5	1.149 (2)
N6—C6	1.148 (2)
N7—C7	1.156 (2)
N8—C8	1.146 (2)

**Table 2:** Hydrogen-bond geometry (Å, °)

*D*—H⋯*A*	*D*—H	H⋯*A*	*D*⋯*A*	*D*—H⋯*A*
O1—H1*A*⋯N7	0.82	2.34	3.143 (2)	167
O1—H1*B*⋯N3	0.82	2.12	2.939 (2)	172
O2—H2*A*⋯N8^i^	0.82	2.33	3.118 (3)	163
O2—H2*B*⋯N4	0.82	2.16	2.970 (2)	170
O3*A*—H3*A*⋯O2^ii^	0.82	2.22	2.971 (3)	153
O3*A*—H3*B*⋯O1	0.82	2.12	2.930 (3)	169
O3*B*—H3*C*⋯O1	0.85	1.97	2.823 (16)	179
O3*B*—H3*D*⋯O2^ii^	0.90	2.11	2.889 (18)	144
O4*A*—H4*A*⋯N6^iii^	0.84	2.11	2.930 (6)	165
O4*A*—H4*B*⋯N5	0.82	2.13	2.870 (4)	151
O4*B*—H4*D*⋯N5	0.82	2.09	2.848 (4)	153

Symmetry codes: (i) 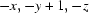; (ii) 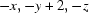; (iii) 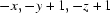.

**Fig. 1.**
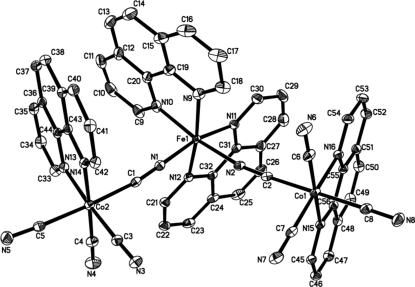
The asymmetric unit of the structure of the title complex showing 50% probability displacement ellipsoids. Water molecules have been omitted for clarity.

**Fig. 2.**
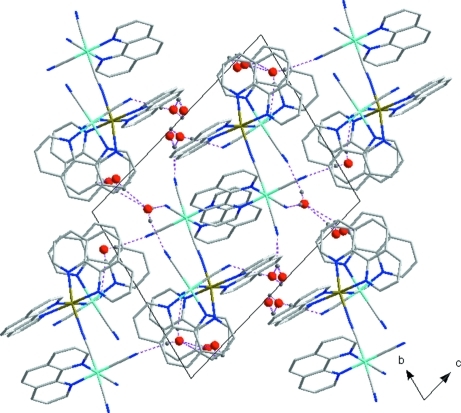
Hydrogen-bonded supramolecular structure of the complex.

**Fig. 3.**
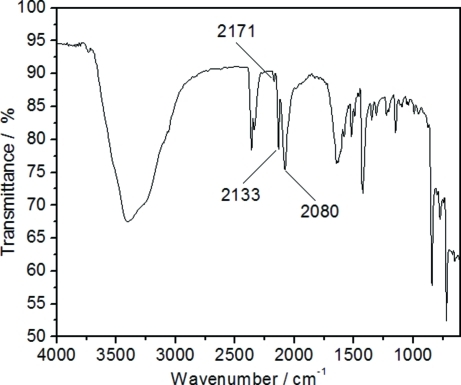
IR spectrum of the title complex.

**Fig. 4.**
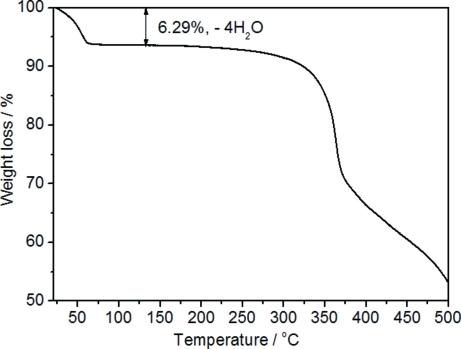
Thermogravimetric curve of the title complex.

## supplementary crystallographic information

### Comment

Cyanide-bridged complexes have been investigated extensively over the past few years due to their excellent properties and potential applications, such as, high *T*_c_ molecular-based magnets (Ferlay *et al.*, 1995; Kosaka *et al.*, 2009), hydrogen storages (Reguera, Balmaseda & Krap *et al.*, 2008; Reguera, Balmaseda & del Castillo *et al.*, 2008), negative thermal expansion materials (Goodwin *et al.*, 2008; Phillips *et al.*, 2008), and so on. Increasing studies have shown that the hydrothermal reaction is a versatile and useful technique to prepare cyanide-bridged complexes, though the majority of synthetic procedures of cyanide-based systems still follow conventional solution routes. Recently, Colacio (Colacio *et al.*, 2003; Rodriguez *et al.*, 2005; Colacio *et al.*, 2005; Rodríguez-Diéguez *et al.*, 2007) and others (Yu *et al.*, 2003; He *et al.*, 2005; Mao *et al.*, 2005; Chen *et al.*, 2006) have shown that cyanide-bridged bimetallic complexes can also be assembled through hydrothermal reactions by using either [*M*(CN)_6_]^3-^ (*M* = Fe^III^, Co^III^) anions as a source of cyanide groups, which act as both reducing agents and bridging ligands.

Bearing this in mind, we introduced CoCl_2_.6H_2_O, 1,10-phenanthroline (phen), and K_3_[Fe(CN)_6_] (or Na_2_[Fe(CN)_5_NO].2H_2_O) into the reaction in order to obtain a Co—Fe bimetallic monometallic complex. It is interesting that a novel cyanide-bridged trinuclear cluster [{Co^III^(phen)(CN)_4_}_2_{Fe^II^(phen)_2_}].4H_2_O, was obtained. It should be noted here that, to the best of our knowledge, the title complex is the first example of a trinuclear cluster prepared by hydrothermal method in the cyanide-based system.

The asymmetric unit of the structure of the title complex is given in Fig. 1. Selected bond lengths and angles are listed in Table 1. Within the neutral [{Co^III^(phen)(CN)_4_}_2_{Fe^II^(phen)_2_}] unit, there are one Fe^II^ center and two Co^III^ centers, with {FeN6} and {CoN2C4} coordination environments, respectively. The Fe center is six-coordinate and adopts a distorted slightly octahedral geometry. Each Fe center is coordinated with two coordinated phen ligands and two bridging cyanide groups in a *cis* arrangement with the angle N1—Fe1—N2 = 89.63 (6)°. The dihedral angle between the planes of chelating phen ligands with the Fe1 atom is *ca* 85°. The mean basal plane is constructed by three N atoms (N9, N11, and N12) from two phen ligands and N1 atom from one bridging cyanide group, while the axial positions are occupied by N10 atom from one phen ligand and N2 atom of the other bridging cyanide group. The geometrical data of the [Fe^II^(phen)_2_(CN)_2_] unit in the title complex are similar to those found for the [Fe^II^(phen)_2_(CN)_2_] unit in the one-dimensional complex [Cu_2_Fe^II^(CN)_4_(phen)_3_]*_n_*.0.5*n*H_2_O (He *et al.*, 2005), the [Fe^II^(bipy)_2_(CN)_2_] unit in the two-dimensional complex [Fe^II^(bipy)_2_(CN)_4_Cu_2_] (Colacio *et al.*, 2003), and three-dimensional complex [Fe^II^(CN)_4_(phen)_2_Cu_2_] (Colacio *et al.*, 2005).

The Co^III^ centers (Co1 and Co2) are both coordinated by two N atoms from one phen ligand, one C atom from one bridging cyanide ligand, and three C atoms from three terminal cyanide ligands. For the Co^III^ centers, the basal plane is formed by two N atoms of one phen ligand and two C atoms of two terminal cyanide groups, while the axial sites are occupied by two C atoms of the other two cyanide groups. As in all other cyanide-bridged complexes, the M—C bond is much shorter than the M—N bond (Table 1). Furthermore, the Co—C—N angles are closed to be linear with the angles spanning from 171° to 179°, which are comparable with those observed for the complexes obtained by hydrothermal methods (Colacio *et al.*, 2003; He *et al.*, 2005; Mao *et al.*, 2005; Colacio *et al.*, 2005), based on [Fe(CN)_6_]^3-^ as the building block.

Thus, cyanide bridges connect one Fe^II^ atom to two Co^III^ atoms in *cis* arrangement, giving rise to a Co^III^_2_Fe^II^ trinuclear cluster with a Fe1 ··· Co1 distance of 5.052 Å and a Fe1 ··· Co2 distance of 5.056 Å. It is noteworthy that the structure of the title complex is distinguished from that of cyanide-based mixed-valence Co^II^/Co^III^ complexes (Halbauer *et al.*, 2008; Guo *et al.*, 2007), and mixed-valence Fe^II^/Fe^III^ (Zhao *et al.*, 2008; Overgaard *et al.*, 2004; Brewer *et al.*, 2007; Xie *et al.*, 2007; Fernández-Armas *et al.*, 2007; Paredes-García *et al.*, 2006) complexes belonging to other systems.

The crystallized water molecules are hydrogen-bonded to each other and terminal cyanide groups. The probable hydrogen bonding interactions are given in Table 2. In addition, weak face-to-face π-*π*interactions between the aromatic rings of adjacent phen ligands from neighboring trinuclear clusters also play important roles in the formation and stabilization of the three-dimensional supramolecular structure (Fig. 2). The distance between two adjacent aromatic ring center is *ca* 3.63 Å.

The IR spectrum (Fig. 3) of the title complex exhibits two strong peaks at 2080 cm^-1^ and 2133 cm^-1^, and one weak peak at 2171 cm^-1^, which indicates the existence of different types of cyanide bridges in the structure. The lower frequencies at 2080 cm^-1^ and 2133 cm^-1^ are reasonably assigned to the terminal cyanide stretching vibrations, while the higher one of 2171 cm^-1^ confirms the presence of bridging cyanide groups.

There is a broad band at the wavenumber range of 3700–2900 cm^-1^ ascribed to the O—H stretching absorption (*ν*_O—H_) in H_2_O molecules. The IR spectrum exhibits characteristic strong bands of the coordinated phen ligands at 1638, 1521, 1425, 844, and 722 cm^-1^ (*δ*_C—H_ benzene ring). The bands at 1521, 1425 and 722 cm^-1^ are shifted from their positions for the free phen ligands (1503, 1420 and 737 cm^-1^), indicating nitrogen coordination. The IR feature has been confirmed by single-crystal X-ray crystallographic analysis.

Thermogravimetric analysis (Fig. 4) is performed to study the thermal stability of the title complex, which shows the title complex loses four crystallized water molecules at above 333 K with a weight loss of 6.29% (Calc. 6.17%). The anhydrous phase loses no further mass up to about 573 K, above which thermal decomposition occurs.

### Experimental

All starting reagents were of analytical grade quality, obtained from commercial sources and used without further purification. A mixture of CoCl_2_.6H_2_O (0.1071 g, 0.45 mmol), 1,10-phenanthroline (phen, 0.0892 g, 0.45 mmol), K_3_[Fe(CN)_6_] (0.1482 g, 0.45 mmol) in a molar ratio of 1:1:1 combined with 10 ml deionized water was stirred for 20 min at room temperature and then transferred into a 25 ml Teflon-lined stainless-steel vessel. The mixture was heated hydrothermally at 413 K for two days under autogenous pressure. Slow cooling of the resulting solution to room temperature afforded dark red, prism-shaped crystals suitable for single-crystal X-ray structure analysis. Yield: 30% (based on Fe). These crystals were separated, washed thoroughly with deionized water and finally with ethanol, and dried. Analysis calculated for C_56_H_40_N_16_O_4_Co_2_Fe: C 57.51, H 3.42, N 19.17%. Found: C  57.46, H 3.35, N 19.12%. EDS (energy dispersive spectrometer): Fe 32.33, Co 67.67. It is of interest that when working under the same hydrothermal conditions, except for using Na_2_[Fe(CN)_5_NO] instead of K_3_[Fe(CN)_6_] as the cyanide source, the same product was obtained (CCDC-732054). From the viewpoint of the mechanism of the formation of the title complex, it is reasonable that free cyanide groups from the dissociation of [Fe(CN)_6_]^3-^ or [Fe(CN)_5_NO]^2-^ might be responsible for the oxidation of the Co center from the original reduction state +II in the precursor CoCl_2_.6H_2_O to the oxidation state +III in the title complex.

### Refinement

All non-H atoms were refined anisotropically. The C(H) atoms of the phen ligand were placed in calculated position (C—H = 0.95 Å) and refined using a riding model, with *U*_iso_(H) = 1.2*U*_eq_(C). The O(H) atoms of the water molecules were located in a difference Fourier map and refined as riding, with *U*_iso_(H) = 1.5*U*_eq_(O). O3 and O4 were both split into two positions (O3A and O3B, and O4A and O4B, respectively) with occupancy of 50% each.

### Crystal data

**Table:**

[Co_2_Fe(CN)_8_(C_12_H_8_N_2_)_4_]·4H_2_O	*Z* = 2
*M**_r_* = 1174.75	*F*(000) = 1200
Triclinic, *P*1	*D*_x_ = 1.521 Mg m^−^^3^
Hall symbol: -P 1	Mo *K*α radiation, λ = 0.71073 Å
*a* = 12.855 (3) Å	Cell parameters from 7618 reflections
*b* = 14.006 (3) Å	θ = 3.2–27.0°
*c* = 16.334 (3) Å	µ = 0.98 mm^−^^1^
α = 72.68 (3)°	*T* = 173 K
β = 82.54 (3)°	Block, dark red
γ = 65.99 (3)°	0.74 × 0.56 × 0.33 mm
*V* = 2564.5 (12) Å^3^	

### Data collection

**Table:**

Rigaku R-AXIS Spider diffractometer	11723 independent reflections
Radiation source: fine-focus sealed tube	10898 reflections with *I* > 2σ(*I*)
graphite	*R*_int_ = 0.040
φ and ω scans	θ_max_ = 27.5°, θ_min_ = 3.1°
Absorption correction: multi-scan (*ABSCOR*; Higashi, 1995)	*h* = −16→16
*T*_min_ = 0.555, *T*_max_ = 0.755	*k* = −18→17
41118 measured reflections	*l* = −21→21

### Refinement

**Table:**

Refinement on *F*^2^	Primary atom site location: structure-invariant direct methods
Least-squares matrix: full	Secondary atom site location: difference Fourier map
*R*[*F*^2^ > 2σ(*F*^2^)] = 0.031	Hydrogen site location: inferred from neighbouring sites
*wR*(*F*^2^) = 0.079	H-atom parameters constrained
*S* = 1.03	*w* = 1/[σ^2^(*F*_o_^2^) + (0.0331*P*)^2^ + 1.5388*P*] where *P* = (*F*_o_^2^ + 2*F*_c_^2^)/3
11723 reflections	(Δ/σ)_max_ = 0.001
726 parameters	Δρ_max_ = 0.44 e Å^−^^3^
0 restraints	Δρ_min_ = −0.70 e Å^−^^3^

### Special details

**Table:**

Geometry. All e.s.d.'s (except the e.s.d. in the dihedral angle between two l.s. planes) are estimated using the full covariance matrix. The cell e.s.d.'s are taken into account individually in the estimation of e.s.d.'s in distances, angles and torsion angles; correlations between e.s.d.'s in cell parameters are only used when they are defined by crystal symmetry. An approximate (isotropic) treatment of cell e.s.d.'s is used for estimating e.s.d.'s involving l.s. planes.
Refinement. Refinement of *F*^2^ against ALL reflections. The weighted *R*-factor *wR* and goodness of fit *S* are based on *F*^2^, conventional *R*-factors *R* are based on *F*, with *F* set to zero for negative *F*^2^. The threshold expression of *F*^2^ > σ(*F*^2^) is used only for calculating *R*-factors(gt) *etc*. and is not relevant to the choice of reflections for refinement. *R*-factors based on *F*^2^ are statistically about twice as large as those based on *F*, and *R*- factors based on ALL data will be even larger.

### Fractional atomic coordinates and isotropic or equivalent isotropic displacement parameters (Å^2^)

**Table:**

	*x*	*y*	*z*	*U*_iso_*/*U*_eq_	Occ. (<1)
Co1	−0.124533 (16)	0.330081 (16)	0.192167 (12)	0.01867 (6)	
Co2	0.211367 (16)	0.613907 (16)	0.329103 (12)	0.01846 (5)	
Fe1	0.272543 (16)	0.259948 (16)	0.270557 (12)	0.01544 (5)	
N1	0.22567 (11)	0.40567 (11)	0.29752 (8)	0.0231 (3)	
N2	0.11105 (11)	0.30592 (11)	0.23131 (8)	0.0225 (3)	
N3	−0.03701 (12)	0.73776 (12)	0.28215 (9)	0.0272 (3)	
N4	0.27633 (15)	0.69837 (14)	0.14501 (10)	0.0416 (4)	
N5	0.22982 (14)	0.80318 (13)	0.37282 (11)	0.0354 (3)	
N6	−0.15698 (12)	0.26931 (14)	0.38441 (9)	0.0357 (4)	
N7	−0.22187 (13)	0.57067 (12)	0.18027 (10)	0.0329 (3)	
N8	−0.35730 (13)	0.34691 (15)	0.15092 (10)	0.0377 (4)	
N9	0.23205 (12)	0.19390 (12)	0.39825 (9)	0.0275 (3)	
N10	0.42921 (12)	0.19929 (12)	0.33001 (10)	0.0288 (3)	
N11	0.31060 (11)	0.13043 (11)	0.21731 (9)	0.0267 (3)	
N12	0.33409 (11)	0.31894 (11)	0.15018 (8)	0.0235 (3)	
N13	0.36947 (11)	0.52399 (10)	0.36591 (8)	0.0204 (2)	
N14	0.17586 (10)	0.55913 (10)	0.45039 (8)	0.0184 (2)	
N15	−0.08549 (10)	0.35289 (10)	0.06914 (8)	0.0191 (2)	
N16	−0.05795 (11)	0.17536 (11)	0.19295 (8)	0.0213 (3)	
C1	0.21160 (12)	0.48801 (13)	0.30657 (9)	0.0204 (3)	
C2	0.02146 (13)	0.31837 (12)	0.21564 (9)	0.0203 (3)	
C3	0.05769 (13)	0.69236 (12)	0.29917 (9)	0.0214 (3)	
C4	0.25134 (14)	0.66576 (14)	0.21445 (10)	0.0269 (3)	
C5	0.22034 (13)	0.73388 (13)	0.35471 (10)	0.0239 (3)	
C6	−0.14966 (13)	0.29636 (14)	0.31107 (10)	0.0250 (3)	
C7	−0.18567 (13)	0.47879 (14)	0.18580 (10)	0.0239 (3)	
C8	−0.27138 (14)	0.34141 (14)	0.16896 (10)	0.0253 (3)	
C9	0.52910 (15)	0.19642 (15)	0.29379 (15)	0.0383 (4)	
H9	0.5351	0.2178	0.2330	0.046*	
C10	0.62613 (16)	0.16242 (16)	0.34347 (19)	0.0520 (6)	
H10	0.6962	0.1614	0.3159	0.062*	
C11	0.62013 (18)	0.13123 (15)	0.43026 (18)	0.0507 (6)	
H11	0.6855	0.1089	0.4635	0.061*	
C12	0.51653 (17)	0.13222 (14)	0.47063 (15)	0.0401 (5)	
C13	0.4999 (2)	0.09982 (15)	0.56160 (15)	0.0511 (6)	
H13	0.5622	0.0768	0.5982	0.061*	
C14	0.3988 (2)	0.10091 (16)	0.59683 (14)	0.0493 (6)	
H14	0.3906	0.0802	0.6574	0.059*	
C15	0.30395 (18)	0.13297 (14)	0.54370 (12)	0.0373 (4)	
C16	0.1974 (2)	0.13174 (15)	0.57587 (12)	0.0439 (5)	
H16	0.1844	0.1117	0.6360	0.053*	
C17	0.11322 (19)	0.15941 (16)	0.52032 (13)	0.0427 (5)	
H17	0.0413	0.1579	0.5414	0.051*	
C18	0.13308 (15)	0.19032 (15)	0.43129 (12)	0.0341 (4)	
H18	0.0734	0.2094	0.3933	0.041*	
C19	0.31693 (15)	0.16514 (13)	0.45389 (11)	0.0280 (3)	
C20	0.42345 (15)	0.16639 (13)	0.41689 (12)	0.0287 (4)	
C21	0.35029 (14)	0.41126 (14)	0.11906 (11)	0.0280 (3)	
H21	0.3371	0.4564	0.1559	0.034*	
C22	0.38586 (15)	0.44472 (15)	0.03462 (11)	0.0317 (4)	
H22	0.3970	0.5110	0.0151	0.038*	
C23	0.40454 (15)	0.38120 (15)	−0.01973 (11)	0.0328 (4)	
H23	0.4278	0.4034	−0.0774	0.039*	
C24	0.38890 (14)	0.28262 (15)	0.01076 (11)	0.0299 (4)	
C25	0.40323 (17)	0.21135 (17)	−0.04085 (12)	0.0396 (4)	
H25	0.4252	0.2300	−0.0993	0.048*	
C26	0.38619 (17)	0.11833 (17)	−0.00829 (13)	0.0414 (5)	
H26	0.3947	0.0736	−0.0445	0.050*	
C27	0.35531 (15)	0.08576 (15)	0.08027 (13)	0.0344 (4)	
C28	0.33953 (17)	−0.01117 (16)	0.11841 (16)	0.0444 (5)	
H28	0.3493	−0.0600	0.0855	0.053*	
C29	0.30996 (17)	−0.03515 (16)	0.20326 (16)	0.0462 (5)	
H29	0.2996	−0.1009	0.2299	0.055*	
C30	0.29509 (15)	0.03833 (15)	0.25071 (14)	0.0374 (4)	
H30	0.2730	0.0214	0.3093	0.045*	
C31	0.34010 (13)	0.15426 (13)	0.13257 (11)	0.0260 (3)	
C32	0.35473 (13)	0.25445 (13)	0.09695 (10)	0.0245 (3)	
C33	0.46540 (14)	0.50782 (14)	0.32018 (11)	0.0265 (3)	
H33	0.4621	0.5467	0.2613	0.032*	
C34	0.57127 (14)	0.43506 (14)	0.35623 (12)	0.0291 (3)	
H34	0.6384	0.4255	0.3218	0.035*	
C35	0.57866 (13)	0.37764 (13)	0.44087 (11)	0.0263 (3)	
H35	0.6505	0.3285	0.4654	0.032*	
C36	0.47828 (13)	0.39246 (12)	0.49103 (10)	0.0225 (3)	
C37	0.47374 (14)	0.33739 (13)	0.57997 (11)	0.0272 (3)	
H37	0.5424	0.2867	0.6088	0.033*	
C38	0.37353 (15)	0.35609 (13)	0.62381 (11)	0.0276 (3)	
H38	0.3733	0.3181	0.6826	0.033*	
C39	0.26799 (13)	0.43218 (12)	0.58304 (10)	0.0219 (3)	
C40	0.16036 (14)	0.45744 (13)	0.62392 (10)	0.0252 (3)	
H40	0.1536	0.4225	0.6827	0.030*	
C41	0.06563 (14)	0.53278 (14)	0.57807 (10)	0.0250 (3)	
H41	−0.0070	0.5516	0.6055	0.030*	
C42	0.07574 (13)	0.58191 (13)	0.49106 (10)	0.0213 (3)	
H42	0.0091	0.6332	0.4600	0.026*	
C43	0.27068 (12)	0.48641 (12)	0.49617 (9)	0.0188 (3)	
C44	0.37582 (12)	0.46686 (12)	0.45018 (10)	0.0194 (3)	
C45	−0.10336 (13)	0.44500 (13)	0.00828 (10)	0.0226 (3)	
H45	−0.1422	0.5119	0.0226	0.027*	
C46	−0.06668 (14)	0.44644 (14)	−0.07650 (10)	0.0269 (3)	
H46	−0.0813	0.5137	−0.1188	0.032*	
C47	−0.00973 (15)	0.35077 (15)	−0.09852 (10)	0.0277 (3)	
H47	0.0158	0.3514	−0.1558	0.033*	
C48	0.01046 (14)	0.25187 (14)	−0.03546 (10)	0.0243 (3)	
C49	0.06927 (15)	0.14634 (15)	−0.04983 (11)	0.0304 (4)	
H49	0.0983	0.1408	−0.1055	0.037*	
C50	0.08422 (15)	0.05469 (14)	0.01403 (11)	0.0303 (4)	
H50	0.1245	−0.0139	0.0026	0.036*	
C51	0.04031 (13)	0.05916 (13)	0.09894 (11)	0.0253 (3)	
C52	0.04915 (15)	−0.03224 (14)	0.16798 (12)	0.0307 (4)	
H52	0.0855	−0.1033	0.1605	0.037*	
C53	0.00447 (15)	−0.01708 (14)	0.24631 (11)	0.0316 (4)	
H53	0.0090	−0.0780	0.2933	0.038*	
C54	−0.04757 (14)	0.08730 (14)	0.25723 (10)	0.0276 (3)	
H54	−0.0765	0.0959	0.3123	0.033*	
C55	−0.01538 (13)	0.16124 (13)	0.11473 (10)	0.0209 (3)	
C56	−0.03016 (12)	0.25769 (12)	0.04762 (9)	0.0200 (3)	
O1	−0.25835 (11)	0.79447 (12)	0.20885 (9)	0.0415 (3)	
H1A	−0.2471	0.7410	0.1926	0.062*	
H1B	−0.1948	0.7814	0.2246	0.062*	
O2	0.28189 (17)	0.81586 (13)	−0.03875 (10)	0.0616 (5)	
H2A	0.2985	0.7640	−0.0584	0.092*	
H2B	0.2886	0.7776	0.0108	0.092*	
O3A	−0.3779 (2)	1.03096 (19)	0.15433 (19)	0.0758 (9)	0.866 (5)
H3A	−0.3618	1.0695	0.1103	0.114*	0.866 (5)
H3B	−0.3378	0.9660	0.1644	0.114*	0.866 (5)
O3B	−0.3120 (16)	1.0178 (13)	0.1828 (13)	0.0758 (9)	0.134 (5)
H3C	−0.2948	0.9503	0.1908	0.114*	0.134 (5)
H3D	−0.3271	1.0645	0.1299	0.114*	0.134 (5)
O4A	0.0980 (4)	0.9375 (4)	0.4824 (3)	0.0666 (17)	0.546 (10)
H4A	0.1018	0.8821	0.5226	0.100*	0.546 (10)
H4B	0.1402	0.9193	0.4430	0.100*	0.546 (10)
O4B	0.1348 (4)	0.9808 (4)	0.4474 (4)	0.0616 (18)	0.454 (10)
H4C	0.0512	1.0005	0.4678	0.092*	0.454 (10)
H4D	0.1514	0.9206	0.4402	0.092*	0.454 (10)

### Atomic displacement parameters (Å^2^)

**Table:**

	*U*^11^	*U*^22^	*U*^33^	*U*^12^	*U*^13^	*U*^23^
Co1	0.01406 (10)	0.02221 (11)	0.01562 (10)	−0.00363 (8)	−0.00056 (7)	−0.00400 (8)
Co2	0.01609 (10)	0.01932 (10)	0.01740 (10)	−0.00382 (8)	0.00002 (7)	−0.00580 (8)
Fe1	0.01103 (9)	0.01723 (10)	0.01583 (10)	−0.00423 (8)	−0.00069 (7)	−0.00288 (8)
N1	0.0201 (6)	0.0255 (7)	0.0227 (6)	−0.0078 (5)	−0.0019 (5)	−0.0061 (5)
N2	0.0178 (6)	0.0213 (6)	0.0221 (6)	−0.0030 (5)	−0.0008 (5)	−0.0032 (5)
N3	0.0231 (7)	0.0308 (7)	0.0236 (7)	−0.0049 (6)	−0.0026 (5)	−0.0084 (6)
N4	0.0442 (10)	0.0446 (10)	0.0260 (8)	−0.0130 (8)	0.0058 (7)	−0.0044 (7)
N5	0.0332 (8)	0.0305 (8)	0.0433 (9)	−0.0082 (7)	−0.0060 (7)	−0.0144 (7)
N6	0.0211 (7)	0.0528 (10)	0.0221 (7)	−0.0059 (7)	0.0024 (5)	−0.0075 (7)
N7	0.0334 (8)	0.0305 (8)	0.0283 (7)	−0.0022 (6)	−0.0074 (6)	−0.0107 (6)
N8	0.0261 (8)	0.0523 (10)	0.0325 (8)	−0.0189 (7)	−0.0042 (6)	−0.0016 (7)
N9	0.0227 (7)	0.0257 (7)	0.0286 (7)	−0.0032 (6)	0.0009 (5)	−0.0089 (6)
N10	0.0185 (6)	0.0246 (7)	0.0447 (8)	−0.0071 (6)	0.0010 (6)	−0.0139 (6)
N11	0.0176 (6)	0.0250 (7)	0.0333 (7)	−0.0084 (6)	−0.0059 (5)	0.0005 (6)
N12	0.0231 (6)	0.0239 (7)	0.0219 (6)	−0.0096 (6)	−0.0034 (5)	−0.0020 (5)
N13	0.0165 (6)	0.0202 (6)	0.0237 (6)	−0.0044 (5)	0.0004 (5)	−0.0090 (5)
N14	0.0176 (6)	0.0186 (6)	0.0194 (6)	−0.0058 (5)	−0.0002 (5)	−0.0075 (5)
N15	0.0160 (6)	0.0218 (6)	0.0177 (6)	−0.0062 (5)	−0.0008 (5)	−0.0043 (5)
N16	0.0187 (6)	0.0221 (6)	0.0202 (6)	−0.0071 (5)	−0.0008 (5)	−0.0024 (5)
C1	0.0153 (7)	0.0261 (8)	0.0169 (6)	−0.0051 (6)	0.0001 (5)	−0.0062 (6)
C2	0.0198 (7)	0.0193 (7)	0.0161 (6)	−0.0027 (6)	0.0010 (5)	−0.0039 (5)
C3	0.0233 (8)	0.0221 (7)	0.0175 (7)	−0.0065 (6)	0.0002 (6)	−0.0070 (6)
C4	0.0241 (8)	0.0267 (8)	0.0250 (8)	−0.0055 (7)	0.0008 (6)	−0.0070 (6)
C5	0.0189 (7)	0.0232 (8)	0.0239 (7)	−0.0033 (6)	−0.0020 (6)	−0.0045 (6)
C6	0.0129 (7)	0.0332 (9)	0.0233 (8)	−0.0035 (6)	0.0009 (5)	−0.0079 (6)
C7	0.0184 (7)	0.0301 (8)	0.0188 (7)	−0.0037 (6)	−0.0032 (6)	−0.0073 (6)
C8	0.0231 (8)	0.0298 (8)	0.0187 (7)	−0.0087 (7)	0.0011 (6)	−0.0032 (6)
C9	0.0210 (8)	0.0286 (9)	0.0652 (13)	−0.0103 (7)	0.0039 (8)	−0.0133 (9)
C10	0.0198 (9)	0.0264 (10)	0.107 (2)	−0.0088 (8)	−0.0075 (10)	−0.0107 (11)
C11	0.0358 (11)	0.0200 (9)	0.0948 (19)	−0.0052 (8)	−0.0312 (11)	−0.0087 (10)
C12	0.0379 (10)	0.0169 (8)	0.0654 (13)	−0.0018 (7)	−0.0244 (9)	−0.0138 (8)
C13	0.0695 (16)	0.0226 (9)	0.0585 (13)	−0.0035 (10)	−0.0400 (12)	−0.0112 (9)
C14	0.0746 (17)	0.0268 (9)	0.0397 (11)	−0.0047 (10)	−0.0236 (11)	−0.0110 (8)
C15	0.0548 (12)	0.0189 (8)	0.0304 (9)	−0.0014 (8)	−0.0074 (8)	−0.0111 (7)
C16	0.0616 (13)	0.0251 (9)	0.0284 (9)	−0.0016 (9)	0.0082 (9)	−0.0090 (7)
C17	0.0432 (11)	0.0311 (10)	0.0373 (10)	−0.0035 (9)	0.0147 (9)	−0.0079 (8)
C18	0.0261 (8)	0.0315 (9)	0.0342 (9)	−0.0045 (7)	0.0066 (7)	−0.0067 (7)
C19	0.0314 (9)	0.0178 (7)	0.0298 (8)	−0.0005 (7)	−0.0057 (7)	−0.0102 (6)
C20	0.0275 (8)	0.0171 (7)	0.0405 (9)	−0.0021 (6)	−0.0099 (7)	−0.0121 (7)
C21	0.0263 (8)	0.0274 (8)	0.0289 (8)	−0.0123 (7)	−0.0026 (6)	−0.0022 (7)
C22	0.0270 (8)	0.0313 (9)	0.0316 (9)	−0.0136 (7)	−0.0022 (7)	0.0029 (7)
C23	0.0247 (8)	0.0381 (10)	0.0242 (8)	−0.0077 (7)	−0.0001 (6)	0.0012 (7)
C24	0.0233 (8)	0.0318 (9)	0.0246 (8)	−0.0024 (7)	−0.0041 (6)	−0.0039 (7)
C25	0.0357 (10)	0.0425 (11)	0.0285 (9)	−0.0012 (9)	−0.0042 (7)	−0.0110 (8)
C26	0.0370 (10)	0.0386 (10)	0.0425 (11)	0.0007 (8)	−0.0117 (8)	−0.0196 (9)
C27	0.0243 (8)	0.0283 (9)	0.0472 (11)	−0.0017 (7)	−0.0107 (7)	−0.0130 (8)
C28	0.0294 (10)	0.0294 (10)	0.0745 (15)	−0.0053 (8)	−0.0110 (10)	−0.0186 (10)
C29	0.0294 (10)	0.0227 (9)	0.0813 (16)	−0.0101 (8)	−0.0108 (10)	−0.0027 (9)
C30	0.0225 (8)	0.0299 (9)	0.0506 (11)	−0.0104 (7)	−0.0086 (8)	0.0056 (8)
C31	0.0183 (7)	0.0248 (8)	0.0305 (8)	−0.0043 (6)	−0.0073 (6)	−0.0043 (6)
C32	0.0187 (7)	0.0253 (8)	0.0245 (7)	−0.0047 (6)	−0.0050 (6)	−0.0035 (6)
C33	0.0217 (8)	0.0288 (8)	0.0276 (8)	−0.0077 (7)	0.0037 (6)	−0.0103 (7)
C34	0.0177 (7)	0.0310 (9)	0.0387 (9)	−0.0051 (7)	0.0043 (7)	−0.0176 (7)
C35	0.0166 (7)	0.0224 (8)	0.0400 (9)	−0.0022 (6)	−0.0036 (6)	−0.0147 (7)
C36	0.0200 (7)	0.0170 (7)	0.0315 (8)	−0.0047 (6)	−0.0044 (6)	−0.0097 (6)
C37	0.0256 (8)	0.0193 (7)	0.0338 (8)	−0.0050 (6)	−0.0110 (7)	−0.0040 (6)
C38	0.0316 (9)	0.0240 (8)	0.0259 (8)	−0.0112 (7)	−0.0083 (7)	−0.0007 (6)
C39	0.0252 (8)	0.0207 (7)	0.0228 (7)	−0.0108 (6)	−0.0033 (6)	−0.0061 (6)
C40	0.0308 (8)	0.0286 (8)	0.0196 (7)	−0.0157 (7)	0.0010 (6)	−0.0060 (6)
C41	0.0237 (8)	0.0302 (8)	0.0239 (7)	−0.0123 (7)	0.0052 (6)	−0.0111 (6)
C42	0.0182 (7)	0.0231 (7)	0.0228 (7)	−0.0063 (6)	0.0010 (6)	−0.0095 (6)
C43	0.0183 (7)	0.0172 (7)	0.0225 (7)	−0.0063 (6)	−0.0018 (5)	−0.0080 (6)
C44	0.0183 (7)	0.0172 (7)	0.0239 (7)	−0.0057 (6)	−0.0016 (6)	−0.0085 (6)
C45	0.0206 (7)	0.0217 (7)	0.0228 (7)	−0.0073 (6)	−0.0028 (6)	−0.0025 (6)
C46	0.0291 (8)	0.0288 (8)	0.0199 (7)	−0.0133 (7)	−0.0021 (6)	0.0013 (6)
C47	0.0306 (8)	0.0367 (9)	0.0178 (7)	−0.0166 (7)	0.0026 (6)	−0.0062 (6)
C48	0.0228 (7)	0.0297 (8)	0.0221 (7)	−0.0113 (7)	0.0019 (6)	−0.0085 (6)
C49	0.0312 (9)	0.0359 (9)	0.0285 (8)	−0.0142 (8)	0.0084 (7)	−0.0167 (7)
C50	0.0279 (8)	0.0287 (8)	0.0360 (9)	−0.0090 (7)	0.0045 (7)	−0.0158 (7)
C51	0.0206 (7)	0.0246 (8)	0.0297 (8)	−0.0076 (6)	−0.0010 (6)	−0.0075 (6)
C52	0.0263 (8)	0.0213 (8)	0.0403 (9)	−0.0064 (7)	−0.0043 (7)	−0.0048 (7)
C53	0.0308 (9)	0.0243 (8)	0.0322 (9)	−0.0098 (7)	−0.0051 (7)	0.0038 (7)
C54	0.0266 (8)	0.0283 (8)	0.0225 (7)	−0.0103 (7)	−0.0014 (6)	0.0009 (6)
C55	0.0176 (7)	0.0226 (7)	0.0210 (7)	−0.0072 (6)	−0.0009 (5)	−0.0044 (6)
C56	0.0169 (7)	0.0220 (7)	0.0206 (7)	−0.0071 (6)	−0.0009 (5)	−0.0053 (6)
O1	0.0304 (7)	0.0427 (8)	0.0432 (8)	−0.0101 (6)	−0.0054 (6)	−0.0036 (6)
O2	0.0890 (13)	0.0424 (9)	0.0356 (8)	−0.0130 (9)	0.0044 (8)	−0.0055 (7)
O3A	0.0557 (17)	0.0548 (12)	0.113 (2)	−0.0134 (12)	0.0160 (14)	−0.0362 (13)
O3B	0.0557 (17)	0.0548 (12)	0.113 (2)	−0.0134 (12)	0.0160 (14)	−0.0362 (13)
O4A	0.074 (3)	0.041 (2)	0.073 (3)	−0.0097 (19)	0.011 (2)	−0.021 (2)
O4B	0.080 (3)	0.034 (2)	0.074 (3)	−0.022 (2)	0.023 (2)	−0.028 (2)

### Geometric parameters (Å, °)

**Table:**

Co1—C6	1.8747 (16)	C23—H23	0.9500
Co1—C7	1.8779 (18)	C24—C32	1.407 (2)
Co1—C2	1.8960 (16)	C24—C25	1.432 (3)
Co1—C8	1.9076 (17)	C25—C26	1.348 (3)
Co1—N15	1.9693 (13)	C25—H25	0.9500
Co1—N16	1.9762 (15)	C26—C27	1.437 (3)
Co2—C3	1.8744 (17)	C26—H26	0.9500
Co2—C4	1.8822 (17)	C27—C28	1.400 (3)
Co2—C5	1.8975 (17)	C27—C31	1.407 (2)
Co2—C1	1.9044 (16)	C28—C29	1.367 (3)
Co2—N13	1.9652 (15)	C28—H28	0.9500
Co2—N14	1.9665 (14)	C29—C30	1.405 (3)
Fe1—N2	2.0365 (14)	C29—H29	0.9500
Fe1—N1	2.0464 (15)	C30—H30	0.9500
Fe1—N12	2.0821 (15)	C31—C32	1.429 (2)
Fe1—N10	2.0845 (16)	C33—C34	1.400 (2)
Fe1—N11	2.0960 (16)	C33—H33	0.9500
Fe1—N9	2.1067 (16)	C34—C35	1.370 (3)
N1—C1	1.144 (2)	C34—H34	0.9500
N2—C2	1.144 (2)	C35—C36	1.410 (2)
N3—C3	1.147 (2)	C35—H35	0.9500
N4—C4	1.147 (2)	C36—C44	1.401 (2)
N5—C5	1.149 (2)	C36—C37	1.434 (2)
N6—C6	1.148 (2)	C37—C38	1.356 (2)
N7—C7	1.156 (2)	C37—H37	0.9500
N8—C8	1.146 (2)	C38—C39	1.434 (2)
N9—C18	1.330 (2)	C38—H38	0.9500
N9—C19	1.364 (2)	C39—C43	1.399 (2)
N10—C9	1.332 (2)	C39—C40	1.410 (2)
N10—C20	1.358 (2)	C40—C41	1.370 (2)
N11—C30	1.328 (2)	C40—H40	0.9500
N11—C31	1.365 (2)	C41—C42	1.397 (2)
N12—C21	1.332 (2)	C41—H41	0.9500
N12—C32	1.362 (2)	C42—H42	0.9500
N13—C33	1.328 (2)	C43—C44	1.423 (2)
N13—C44	1.365 (2)	C45—C46	1.401 (2)
N14—C42	1.3302 (19)	C45—H45	0.9500
N14—C43	1.362 (2)	C46—C47	1.372 (3)
N15—C45	1.327 (2)	C46—H46	0.9500
N15—C56	1.360 (2)	C47—C48	1.405 (2)
N16—C54	1.331 (2)	C47—H47	0.9500
N16—C55	1.361 (2)	C48—C56	1.403 (2)
C9—C10	1.411 (3)	C48—C49	1.436 (2)
C9—H9	0.9500	C49—C50	1.352 (3)
C10—C11	1.355 (4)	C49—H49	0.9500
C10—H10	0.9500	C50—C51	1.437 (2)
C11—C12	1.404 (3)	C50—H50	0.9500
C11—H11	0.9500	C51—C55	1.401 (2)
C12—C20	1.410 (2)	C51—C52	1.407 (2)
C12—C13	1.433 (3)	C52—C53	1.373 (3)
C13—C14	1.347 (4)	C52—H52	0.9500
C13—H13	0.9500	C53—C54	1.395 (3)
C14—C15	1.428 (3)	C53—H53	0.9500
C14—H14	0.9500	C54—H54	0.9500
C15—C16	1.407 (3)	C55—C56	1.423 (2)
C15—C19	1.410 (2)	O1—H1A	0.8200
C16—C17	1.360 (3)	O1—H1B	0.8200
C16—H16	0.9500	O2—H2A	0.8200
C17—C18	1.409 (3)	O2—H2B	0.8200
C17—H17	0.9500	O3A—H3A	0.8199
C18—H18	0.9500	O3A—H3B	0.8199
C19—C20	1.428 (3)	O3B—H3C	0.8505
C21—C22	1.399 (2)	O3B—H3D	0.9027
C21—H21	0.9500	O4A—H4A	0.8422
C22—C23	1.371 (3)	O4A—H4B	0.8174
C22—H22	0.9500	O4B—H4C	1.0307
C23—C24	1.409 (3)	O4B—H4D	0.8225
			
C6—Co1—C7	90.48 (8)	N12—C21—C22	122.94 (17)
C6—Co1—C2	87.01 (7)	N12—C21—H21	118.5
C7—Co1—C2	90.57 (7)	C22—C21—H21	118.5
C6—Co1—C8	92.80 (7)	C23—C22—C21	119.48 (17)
C7—Co1—C8	89.57 (8)	C23—C22—H22	120.3
C2—Co1—C8	179.77 (7)	C21—C22—H22	120.3
C6—Co1—N15	174.69 (6)	C22—C23—C24	119.38 (16)
C7—Co1—N15	93.95 (7)	C22—C23—H23	120.3
C2—Co1—N15	90.01 (6)	C24—C23—H23	120.3
C8—Co1—N15	90.17 (7)	C32—C24—C23	117.35 (16)
C6—Co1—N16	92.28 (7)	C32—C24—C25	118.53 (17)
C7—Co1—N16	177.21 (6)	C23—C24—C25	124.11 (17)
C2—Co1—N16	89.20 (7)	C26—C25—C24	121.49 (18)
C8—Co1—N16	90.68 (7)	C26—C25—H25	119.3
N15—Co1—N16	83.28 (6)	C24—C25—H25	119.3
C3—Co2—C4	88.80 (8)	C25—C26—C27	121.08 (18)
C3—Co2—C5	91.73 (7)	C25—C26—H26	119.5
C4—Co2—C5	89.44 (8)	C27—C26—H26	119.5
C3—Co2—C1	91.83 (7)	C28—C27—C31	117.43 (19)
C4—Co2—C1	91.26 (7)	C28—C27—C26	123.79 (19)
C5—Co2—C1	176.38 (6)	C31—C27—C26	118.78 (17)
C3—Co2—N13	176.12 (6)	C29—C28—C27	119.58 (19)
C4—Co2—N13	94.35 (7)	C29—C28—H28	120.2
C5—Co2—N13	90.56 (7)	C27—C28—H28	120.2
C1—Co2—N13	85.85 (6)	C28—C29—C30	119.40 (18)
C3—Co2—N14	93.37 (7)	C28—C29—H29	120.3
C4—Co2—N14	177.57 (6)	C30—C29—H29	120.3
C5—Co2—N14	89.40 (6)	N11—C30—C29	122.93 (19)
C1—Co2—N14	89.76 (6)	N11—C30—H30	118.5
N13—Co2—N14	83.52 (6)	C29—C30—H30	118.5
N2—Fe1—N1	89.63 (6)	N11—C31—C27	123.07 (16)
N2—Fe1—N12	95.57 (6)	N11—C31—C32	117.09 (15)
N1—Fe1—N12	91.20 (6)	C27—C31—C32	119.84 (16)
N2—Fe1—N10	170.46 (6)	N12—C32—C24	123.00 (15)
N1—Fe1—N10	91.63 (6)	N12—C32—C31	116.79 (14)
N12—Fe1—N10	93.86 (6)	C24—C32—C31	120.21 (16)
N2—Fe1—N11	84.52 (6)	N13—C33—C34	121.87 (16)
N1—Fe1—N11	168.24 (5)	N13—C33—H33	119.1
N12—Fe1—N11	79.27 (6)	C34—C33—H33	119.1
N10—Fe1—N11	95.84 (6)	C35—C34—C33	120.36 (15)
N2—Fe1—N9	92.00 (6)	C35—C34—H34	119.8
N1—Fe1—N9	88.78 (6)	C33—C34—H34	119.8
N12—Fe1—N9	172.44 (5)	C34—C35—C36	119.16 (15)
N10—Fe1—N9	78.58 (7)	C34—C35—H35	120.4
N11—Fe1—N9	101.58 (6)	C36—C35—H35	120.4
C1—N1—Fe1	171.17 (13)	C44—C36—C35	116.95 (15)
C2—N2—Fe1	170.94 (13)	C44—C36—C37	118.12 (15)
C18—N9—C19	117.69 (16)	C35—C36—C37	124.93 (15)
C18—N9—Fe1	128.98 (13)	C38—C37—C36	121.37 (15)
C19—N9—Fe1	112.69 (12)	C38—C37—H37	119.3
C9—N10—C20	118.00 (16)	C36—C37—H37	119.3
C9—N10—Fe1	128.11 (14)	C37—C38—C39	121.21 (15)
C20—N10—Fe1	113.72 (11)	C37—C38—H38	119.4
C30—N11—C31	117.58 (16)	C39—C38—H38	119.4
C30—N11—Fe1	129.09 (14)	C43—C39—C40	116.81 (14)
C31—N11—Fe1	112.63 (11)	C43—C39—C38	118.24 (15)
C21—N12—C32	117.82 (14)	C40—C39—C38	124.95 (15)
C21—N12—Fe1	128.64 (12)	C41—C40—C39	119.40 (14)
C32—N12—Fe1	113.47 (11)	C41—C40—H40	120.3
C33—N13—C44	118.25 (14)	C39—C40—H40	120.3
C33—N13—Co2	129.55 (11)	C40—C41—C42	120.06 (15)
C44—N13—Co2	112.08 (10)	C40—C41—H41	120.0
C42—N14—C43	118.18 (13)	C42—C41—H41	120.0
C42—N14—Co2	129.60 (11)	N14—C42—C41	122.02 (15)
C43—N14—Co2	112.22 (10)	N14—C42—H42	119.0
C45—N15—C56	118.39 (13)	C41—C42—H42	119.0
C45—N15—Co1	129.36 (11)	N14—C43—C39	123.49 (14)
C56—N15—Co1	112.23 (10)	N14—C43—C44	115.95 (13)
C54—N16—C55	117.99 (14)	C39—C43—C44	120.55 (14)
C54—N16—Co1	129.90 (12)	N13—C44—C36	123.41 (14)
C55—N16—Co1	112.10 (11)	N13—C44—C43	116.08 (13)
N1—C1—Co2	171.05 (13)	C36—C44—C43	120.51 (14)
N2—C2—Co1	176.64 (14)	N15—C45—C46	121.92 (15)
N3—C3—Co2	178.00 (15)	N15—C45—H45	119.0
N4—C4—Co2	178.90 (17)	C46—C45—H45	119.0
N5—C5—Co2	176.80 (15)	C47—C46—C45	120.05 (15)
N6—C6—Co1	174.75 (15)	C47—C46—H46	120.0
N7—C7—Co1	178.49 (16)	C45—C46—H46	120.0
N8—C8—Co1	176.31 (15)	C46—C47—C48	119.35 (15)
N10—C9—C10	121.6 (2)	C46—C47—H47	120.3
N10—C9—H9	119.2	C48—C47—H47	120.3
C10—C9—H9	119.2	C56—C48—C47	116.96 (15)
C11—C10—C9	120.5 (2)	C56—C48—C49	118.09 (15)
C11—C10—H10	119.8	C47—C48—C49	124.96 (15)
C9—C10—H10	119.8	C50—C49—C48	121.51 (15)
C10—C11—C12	119.47 (19)	C50—C49—H49	119.2
C10—C11—H11	120.3	C48—C49—H49	119.2
C12—C11—H11	120.3	C49—C50—C51	121.16 (16)
C11—C12—C20	116.9 (2)	C49—C50—H50	119.4
C11—C12—C13	124.7 (2)	C51—C50—H50	119.4
C20—C12—C13	118.4 (2)	C55—C51—C52	117.22 (15)
C14—C13—C12	122.14 (19)	C55—C51—C50	118.22 (15)
C14—C13—H13	118.9	C52—C51—C50	124.56 (16)
C12—C13—H13	118.9	C53—C52—C51	118.91 (16)
C13—C14—C15	120.5 (2)	C53—C52—H52	120.5
C13—C14—H14	119.8	C51—C52—H52	120.5
C15—C14—H14	119.8	C52—C53—C54	120.35 (16)
C16—C15—C19	117.28 (18)	C52—C53—H53	119.8
C16—C15—C14	123.6 (2)	C54—C53—H53	119.8
C19—C15—C14	119.1 (2)	N16—C54—C53	122.09 (16)
C17—C16—C15	119.55 (18)	N16—C54—H54	119.0
C17—C16—H16	120.2	C53—C54—H54	119.0
C15—C16—H16	120.2	N16—C55—C51	123.43 (15)
C16—C17—C18	119.7 (2)	N16—C55—C56	116.02 (14)
C16—C17—H17	120.1	C51—C55—C56	120.55 (14)
C18—C17—H17	120.1	N15—C56—C48	123.33 (14)
N9—C18—C17	122.64 (19)	N15—C56—C55	116.23 (13)
N9—C18—H18	118.7	C48—C56—C55	120.44 (14)
C17—C18—H18	118.7	H1A—O1—H1B	101.9
N9—C19—C15	123.11 (17)	H2A—O2—H2B	92.6
N9—C19—C20	116.68 (15)	H3A—O3A—H3B	115.5
C15—C19—C20	120.20 (17)	H3C—O3B—H3D	121.5
N10—C20—C12	123.52 (18)	H4A—O4A—H4B	109.9
N10—C20—C19	116.86 (15)	H4C—O4B—H4D	102.0
C12—C20—C19	119.60 (18)		

### Hydrogen-bond geometry (Å, °)

**Table:**

*D*—H···*A*	*D*—H	H···*A*	*D*···*A*	*D*—H···*A*
O1—H1A···N7	0.82	2.34	3.143 (2)	167
O1—H1B···N3	0.82	2.12	2.939 (2)	172
O2—H2A···N8^i^	0.82	2.33	3.118 (3)	163
O2—H2B···N4	0.82	2.16	2.970 (2)	170
O3A—H3A···O2^ii^	0.82	2.22	2.971 (3)	153
O3A—H3B···O1	0.82	2.12	2.930 (3)	169
O3B—H3C···O1	0.85	1.97	2.823 (16)	179
O3B—H3D···O2^ii^	0.90	2.11	2.889 (18)	144
O4A—H4A···N6^iii^	0.84	2.11	2.930 (6)	165
O4A—H4B···N5	0.82	2.13	2.870 (4)	151
O4B—H4D···N5	0.82	2.09	2.848 (4)	153

Symmetry codes: (i) −*x*, −*y*+1, −*z*; (ii) −*x*, −*y*+2, −*z*; (iii) −*x*, −*y*+1, −*z*+1.
